# The Novel Coronavirus – Latest Findings

**DOI:** 10.1111/1751-7915.13592

**Published:** 2020-05-03

**Authors:** Harald Brüssow

**Affiliations:** ^1^ Department of Biosystems Laboratory of Gene Technology KU Leuven Leuven Belgium

## Abstract

What was initially a lung infection epidemic in the metropolitan area of Wuhan followed by a now contained extension to mainland China has now spread to all continents as a major pandemic with current hotspots in Europe and the USA. This minireview is an update of an earlier report on this novel coronavirus infection (Brüssow, 2020, *Microb Biotech* 13, 607). I am now summarizing the research literature published between end of February to mid‐April 2020.

## COVID‐19 by numbers

The COVID‐19 pandemic presents a highly dynamic picture, both with respect to cases of infection and geographical spread. As of April 10, the time of writing this review, and based on data from the Johns Hopkins University (https/bda7594740fd40299423467b48e9ecf6://gisanddata.maps.arcgis.com/apps/opsdashboard/index.html#/bda7594740fd40299423467b48e9ecf6), it has reached all continents and hotspots of infection have developed in Europe and the USA that outnumber the original focus of infection in China, where the epidemic started end of last year. Worldwide 1.6 million confirmed cases were counted (April 19: 2.3 million). Numerically, the USA leads the field with 466’000 cases, followed by Spain (153k; k = 1000), Italy (143k), France (118k) and Germany (118k), all reporting more cases than China (83k). Then comes UK and Iran (65k each), Turkey (42k), and even small countries are reporting high numbers (Belgium and Switzerland with 24k each). The death toll is, with 96’000 COVID‐19 victims (April 19: 161’000), substantial; Italy and Spain, with 18k and 15k deaths, have had a disproportionately large share, followed by France and the UK. At the moment New York City reports more COVID‐19 deaths (13k on April 19) than China.

## Protection of healthcare personnel

### Preparedness

Despite reports of a strong operational readiness against health emergencies in half of the evaluated countries (Kandel *et al* *.*, 2020), the evolving pandemic has hit the health systems very hard, even in industrialized countries. The lack of personal protective equipment, and of diagnostic tests, put doctors and nurses, in the initial phases of the pandemic, at substantial risk of infection which showed our lack of preparedness (Horton, 2020). By early March, 3300 healthcare workers in China were infected, and 22 died. An Italian survey showed that 20% of healthcare workers were infected (Anonymus, 2020). As health personnel is of critical importance for the affected populations, and can only be partially replaced (e.g. by veterinary doctors in France offering to help out, and by the recruitment of retired health personnel), their protection must be a primary concern.

### Virus stability

The transmission potential of the novel coronavirus cannot be explained by a greater tenacity of the virus, compared to the SARS virus. In aerosols, infectious titre dropped by 10‐fold within 3 h; on plastic, median half‐life was 7 h; but less on cardboards (van Doremalen *et al.*, 2020). For coronavirus, disinfection with ethanol or propanol (85–95%) is effective after 30 s; followed by sodium hypochlorite (0.2%), while chlorhexidine is inefficient (Kampf *et al* *.*, 2020).

### Masks

Quantitative microbial risk assessment data are still lacking for SARS‐CoV‐2, but when using data from Middle East respiratory syndrome (MERS) coronavirus, the daily mean risk of infection was high for nurses and doctors in close bodily contact with the patient, but lower for family visitors and patients sharing the same room. Respiratory masks reduced the infection risks for nurses by > 90%, while increasing the air exchange by ventilation facilitated a 58% risk reduction for visitors and patients sharing the room (Cho *et al*., 2016).

There is only limited evidence for the efficiency of surgical masks against seasonal influenza infection in healthcare personnel, while some evidence exists for mask wearing by infected patients to protect healthcare professionals (Bunyan *et al.*, 2013). For the general public, there is not enough evidence to prove that wearing a surgical mask significantly reduces a healthy person’s risk of becoming infected while wearing it (German Health Ministry, CDC, WHO). While evidence for efficacy of face masks is scarce, evidence is likewise lacking that it is of no use (Feng *et al* *.*, 2020). Some support comes from proxy data. Viral shedding in droplets, and in aerosol, was determined in exhaled breath and coughs from children and adults suffering from the common cold. The subjects were naturally infected with seasonal coronavirus, influenza virus and rhinovirus. Wearing a surgical face mask reduced coronavirus titres, as determined by RT‐PCR, both in droplets and in aerosols; while for influenza virus, masks decreased only viral titres in droplets, and for rhinovirus, the mask had no effect on virus excretion (Leung *et al* *.*, 2020).

### Sampling

Virus detection is made with nasopharyngeal swabbing, which might cause the patient to sneeze, exposing the healthcare worker to infection. Throat swabbing is easier, but viral titres are lower (Zou *et al* *.*, 2020), which leads to a lower sensitivity of the diagnostic test. Early morning saliva coughed up by clearing the throat after wake‐up, sampled by the test person and sent to a diagnostic centre is more sure. Viral titres paralleled those in endotracheal aspirates, albeit at a 10‐fold lower viral titre (To *et al* *.*, 2020).

### Triage

Models have been developed to decongest hospitals when the number of infected persons suddenly increases in an epidemic emergency. For mild and moderate COVID‐19 cases not in need of traditional hospital care, a novel hospital type was developed in China. Public buildings such as stadiums, exhibition centres, factories or warehouses were transformed into shelter hospitals. Construction time was 1–2 days, 12’000 infected subjects found shelter at 19 locations in Wuhan. Due to a low health care to patient ratio, the maintenance cost and staff need was low and these ‘Fangcang’ shelter hospitals could return to their original function after the emergency. Infected patients were thus separated from their uninfected families, received basic medical care, medication and food. Cases with worsening symptoms (13%) were transferred to higher level hospitals for more comprehensive care and recovered patients were discharged in a controlled manner, reducing virus transmission into the community and protecting hospitals from patient overflow (Chen *et al* *.*, 2020d).

Italy became a hotspot of the COVID‐19 epidemic in Europe, pushing the healthcare system to a breaking point. In Milan, an emergency medical system was developed, where patients could call a response team that asks the patients about epidemiological risk factors, and about the severity of respiratory symptoms. According to an algorithm, the response team coordinates transport to a hospital by an ambulance, or opts for home isolation and domicile virus testing (Spina *et al* *.*, 2020).

### Telemedicine

In the United States, 29% of the physicians are older than 55 years. This experienced medical personnel also represents a vulnerable population for severe infections. Instead of being at the front line, this personnel might be better used for advising younger staff and helping in decision‐making (Buerhaus *et al* *.*, 2020). Telemedicine could represent a means to protect doctors in family practices, allowing the control of non‐severe COVID‐19 patients who remain at home or to ensure the follow‐up of stable patients after early hospital discharge. The legal and payment modes for commercial videoconference solutions, with end‐to‐end encryption, are currently being discussed (Keesara *et al.*, 2020). Robotics should be developed for disinfection of high‐risk, high‐touch areas; for diagnostic sample analysis; and for the surveillance of quarantine compliance; and for screening of subjects with elevated body temperature (Yang *et al* *.*, 2020a).

## Clinical observations

### China: adults

Early clinical experience with COVID‐19 patients has been collected by Chinese physicians: 45% of patients from Wuhan reached clinical and radiological remission 10 days after hospitalization. Patients with a protracted disease course were elderly males with comorbidity, showing shortness of breath and the need for mechanical ventilation (Mo *et al* *.*, 2020). In Shanghai, 86% of patients were discharged after a median of 16 days of hospitalization and were virus‐negative after 12 days. All showed initial radiological evidence of pneumonia; 65% showed deterioration after 3 days, followed by an improvement in 95% after 6 days; 9% needed intensive care, and 1% died (Chen *et al* *.*, 2020b). The role of computer tomography (CT) in case management is not yet clear, since after 2 days of symptomatic disease 56% had clear lungs by CT while also asymptomatic infected subjects could show CT lung abnormalities (Chua *et al* *.*, 2020). Notably, 11% of patients showed gut symptoms (diarrhoea > vomiting) and were more severely affected than patients without diarrhoea (Jin et al., 2020).

### China: children

In Wuhan, a large number of children with exposure to COVID‐19 cases were followed: 12% became infected, but the clinical course was mild; 16% showed even no symptoms. Fever developed in 42% of them. Intubation was needed in three children with comorbidity, one child died (Lu *et al* *.*, 2020). In mainland China, 5% of cases were children. Half of them showed mild, or no, upper respiratory tract symptoms. Half had mild pneumonia. Dry cough and abnormal laboratory values were rare compared to adults. Virus excretion was also seen in asymptomatic cases, but was of shorter duration than in pneumonia cases (Qiu *et al* *.*, 2020).

### China: pregnant women

Pregnant women from Wuhan with a confirmed COVID‐19 infection showed similar clinical signs as non‐pregnant women. Their infants, delivered by caesarian section, remained uninfected; amniotic fluid, cord blood and breastmilk from these women were virus‐negative (Chen *et al* *.*, 2020a). In a second study from Wuhan, pregnant women showed mild pneumonia with fever as main symptom. Healthy children were born by caesarian section. One neonate showed a virus‐positive throat swab 36 h after birth, but developed only minor symptoms which resolved (Yu *et al* *.*, 2020). In a third study, infants born by caesarian section to infected mothers showed no symptoms, were negative for the virus in nasopharynx and displayed passive IgG antibodies. Surprisingly, two infants also showed specific IgM antibodies to the virus (Zeng *et al* *.*, 2020).

### China: deaths

Crude case fatality rates (CFR) of 3.7% were reported for mainland China. If milder cases are incorporated into that calculation, CFR were reduced to 1.4% for COVID‐19 patients, which is still substantially higher than that of the 2009 H1N1 influenza pandemic. CFR showed a strong age dependency, with 6.4% for > 60‐year‐old, and 13.4% for > 80‐year‐old patients. Time from onset of symptoms to death was 19 days (Verity *et al* *.*, 2020). In Wuhan, hospital data from patients with definitive outcome of either death or hospital discharge were retrospectively analysed. Non‐survivors differed from survivors for comorbidity, specifically coronary heart disease (24 vs. 1%), hypertension (48% vs. 23%) and diabetes. An increased blood chemistry parameter of thrombosis (d‐dimer) was highly correlated with death. Sepsis was a common complication, but patients showed no bacterial co‐pathogen at admission (Zhou *et al* *.*, 2020). In another study from Wuhan, 62% of patients requiring intensive care had died after a month. Receiving mechanical ventilation and developing acute respiratory distress syndrome (ARDS) predicted death (Yang *et al* *.*, 2020b).

### International death rates

Very different CFR are reported for different countries: Italy 7.7, Iran and Spain 5.7, China 4.0, France and USA 2.4, South Korea 0.9, Switzerland 0.6, Germany 0.2% (status of March 16). These differences must be interpreted with caution, since there is no uniform system for calculating these rates (Lazzarini and Putoto, 2020). The high CFR in Italy might result from a combination of several factors. One is differences in testing the population, affecting the denominator of CFR calculation. After becoming a public health emergency, only ill patients were tested in Italy, while in Korea a much wider range of contacts were tested, and asymptomatic infections were also included into the denominator, thus diluting the death rate (Onder *et al* *.*, 2020). In Korea, CFR differed for males (1.1%) and females (0.4%), for < 40‐year‐olds (0.1%) and > 80‐year‐olds (6%) (Shim *et al* *.*, 2020).

## Therapy trials

### Lopinavir

The repurposing of available antiviral drugs, such as the HIV protease inhibitor lopinavir, is a priority. Clinicians from Wuhan conducted an open‐label clinical trial against controls with about 100 patients per arm. No major improvement was seen in the lopinavir group. The negative outcome might be due to the late drug application in severely affected patients (mortality was 25% in the control group). However, since no effect on viral shedding was seen, it was concluded that lopinavir does not inhibit SARS‐CoV‐2 replication *in vivo* (Baden and Rubin, 2020; Cao *et al* *.*, 2020).

### Plasma

Five severe COVID‐19 patients on mechanical ventilation were treated with plasma from convalescent patients. After transfusion, fever and viral load decreased, oxygenation ameliorated, three patients could be discharged, and two are in stable condition. Definitive conclusions cannot be drawn from this small study due to the lack of controls and concomitant treatment with antivirals (Shen *et al* *.*, 2020a).

### Chloroquine

A small and poorly controlled French trial reported viral clearance at day 6 in 100% of COVID‐19 patients treated with hydroxychloroquine and azithromycin, compared to 57% treated with hydroxychloroquine alone, and 13% in controls (Gautret *et al* *.*, 2020). Other French researchers noted that no acute viral infection has been successfully treated with chloroquine in humans in the past (Touret and de Lamballerie, 2020), and even more severe viral infection was seen in chloroquine‐treated animal model for dengue fever (Guastalegname and Vallone, 2020).

## Epidemiology

### China

Of the 70’000 cases reported by Feb 17 in China, 59% occurred in Wuhan, 24% in Hubei, the province around Wuhan and only 17% in the other provinces of China. The epidemic in mainland China was a mixture of case importation, which was dominant in the early phase, and local transmission, which dominated the later phase. Due to public health measures, the epidemic in mainland China was self‐sustained for only 3 weeks, with basic reproduction numbers R not exceeding 1.7 and then quickly falling below 1 (Zhang *et al* *.*, 2020b). By March 18, no local transmitted cases were reported in China (asymptomatic infections were not registered). Restaurants, shops, schools and factories are reopening while maintaining social distancing measures. Temperature measurements of workers at factory gates and of commuters at subway entrances are installed, together with routine virus testing in all fever patients to prevent secondary flares of the epidemic in China by imported infections (Normile, 2020).

### Mathematical models

Without control measures, the coronavirus epidemic led, in a model calculation within the first 50 days, to 740,000 infections in China. It was deduced that either the travel ban out of Wuhan or the emergence measures (school closure, case and contact isolation, travel restrictions) in mainland China could have reduced this number to 200 000 cases. The combination of both measures was responsible for the lesser toll of an estimated 30 000 cases in mainland China by Feb. 19 (Tian *et al* *.*, 2020). Indeed, the spatial and temporal analysis of the Chinese epidemic showed that until Feb 10, the early epidemic in mainland China was well predicted according to the volume of infected human movement out of Wuhan alone. With sanitary measures taken in Wuhan, this correlation decreased and after the travel ban, local spread and public health counter‐measures determined the epidemic course in mainland China (Kraemer *et al* *.*, 2020). According to a model from the London School of Hygiene, measures to reduce the mixing of the population (school closure, home confinement of the elderly, social distancing at work) have the potential to delay and flatten the epidemic course, allowing the health system to cope with hospitalizations. It is important to relax the restrictions stepwise to avoid emergence of secondary epidemics. With social restrictions, transmission shifts to home clusters and to healthcare professionals (Prem *et al* *.*, 2020).

With 100 undetected, imported cases and no intervention 280’000 cases of infected persons would be expected in Singapore after 80 days, assuming a low R value (R = 1.5). With R = 2.5, 1.2 mio infections would be expected in this model calculation. With the quarantine of the infected person and of family members, school closures, workplace distancing (50% of workforce to work from home for 2 weeks) or all measures combined, the corresponding numbers of infected people would be 15’000, 10’000, 4’000 and 1’800 respectively (at R = 1.5). But at R = 2.5, even the combined measures could only decrease the number of cases of infection to 250’000. Quarantine becomes ineffective if high rates of asymptomatic infections occur (Koo *et al* *.*, 2020).

A model from Imperial College London predicted 500 000 and 2.1 million deaths in UK and USA, respectively, by May/June if no action was to be taken. These predictions changed the government policy with respect to containment measures. The model predicts stepwise decreases of the basic reproduction number of the virus from R = 3.5 to 3.0 by social distancing; to 2.5 by school closure; and to close to 1 by public lockdown (Adam, 2020).

### Super‐spreaders

From three infection clusters in Singapore (a tourist group, a business meeting and a church attendance), incubation times of 4 days and a serial interval for transmission of 3–8 days were determined. Few individuals transmitted the virus to several contacts, while the majority of cases did not infect contacts. Transmission was associated with physical contact (Pung *et al* *.*, 2020). The COVID‐19 epidemic seems to be accelerated by super‐spreaders. The causes are unclear except that transmission rates are much higher in closed settings than in open‐air ones (Frieden and Lee, 2020). The Korean epidemic (6200 cases, 42 death on March 8) was dominated by four clusters. A single church cluster represented 55% of the Korean cases and was seeded by a single super‐spreader (Shim *et al* *.*, 2020). Not all cases are so infectious. A US citizen became virus‐positive on return from Wuhan. She infected her husband, but none of nearly 400 contacts, half of them in the community, half of them in health care (Ghinai *et al* *.*, 2020).

### International spread

US epidemiologists evaluated the effect of the travel ban in Wuhan. A 90% travel reduction delayed virus transmission to mainland China by 1 week, but did not cause case reduction if not combined with local hygiene measures. The impact of travel restriction from Wuhan was internationally greater, but epidemic growth resumed after 2–3 weeks, since too many infected travellers remained undetected and had already seeded new epidemics (Chinazzi *et al.*, 2020). Crossing a critical threshold would have occurred on January 25 and January 28 without travel restriction and with travel restrictions respectively. A major problem with airport control was that 64% of the infected travellers were pre‐symptomatic on arrival (Wells *et al* *.*, 2020). Epidemiologists calculated from air transport data that the risk of case importation into Africa was ten‐fold lower than into Europe. Egypt, Algeria and South Africa were high‐risk countries. When calculating a preparedness and vulnerability index, an epidemic would have the greatest impact on Nigeria (Gilbert *et al* *.*, 2020). The first infections occurred in Europe between January 16–24 by a group of 30 tourists from Wuhan visiting Italy, Switzerland and France. Five of them developed mild disease during travel. They infected 1 out of 40 persons in a high‐risk category, but none out of 216 low‐risk contacts (Olsen *et al* *.*, 2020).

### Mass gatherings

Mass movement, as in the cases of the Chinese and the Iranian New Year celebrations, is a motor of epidemic spread. In addition to social distancing policies, large screening programs and quarantine measures, the most effective single measure in China was the prolongation of the holidays to two weeks which allowed the travellers to develop the disease while still being with the host family (Chen *et al* *.*, 2020c). In contrast, Iran allowed pilgrimage to Qom and experienced a large regional outbreak (Ebrahim and Memish, 2020). School closures are justified by the high surface density of subjects (3–5 m^2^/child) compared to offices (18 m^2^/person) and homes (36 m^2^/person) (US data). School closure can, however, also increase mortality via the immobilization of healthcare personnel without alternate child care available (Bayham and Fenichel, 2020). Shift work should be considered, to increase social distance, where home office is not possible; funerals have to be regulated; and circulation of persons has to be restricted (Ebrahim *et al* *.*, 2020).

### Cruise ship

Interesting epidemiological insights were obtained from particular epidemiological settings. On a cruise ship, a single infected tourist seeded an epidemic which infected 600 of the 3700 people on board. The kinetics of the case development indicated that each infected passenger infected 11 others. When infected passengers were confined to their cabins, the transmission rate dropped 3 days later. The contacts by the cabin crew then maintained a low infection rate until the epidemic died out 10 days later. Overall, 67% of infections were asymptomatic. Surprisingly, more of the older infected passengers remained asymptomatic than younger passengers (43–60% of those > 50 years vs. <28% for the younger passengers) (Mizumoto and Chowell, 2020).

### Contact‐tracing App

When analysing possible transmission routes by symptomatic, pre‐symptomatic, or asymptomatic infected subjects or environmental transmission by fomites, model builders realized that SARS‐CoV‐2 spread is too fast to be contained by manual contact tracing. They propose a contact‐tracing App that warns people who came into close contact over a critical time to a person becoming virus‐positive or symptomatic. While raising ethical problems, such an App used in an early epidemic situation could avoid later lockdowns (Ferretti et al., 2020).

## Virology

### Virus origin

Chinese SARS‐CoV‐2 isolates are so similar in their genome sequence that a single introduction event must be considered. Whether this introduction comes directly from an animal source is less clear. SARS‐CoV‐2 is separated from its closest relative, a bat virus sharing 96% sequence identity, by 20 years of sequence evolution. A coronavirus was also isolated from the lungs of two diseased pangolins, sharing up to 92% nucleotide sequence identity with SARS‐CoV‐2, but with a better binding to the human ACE‐2 virus receptor than the bat virus (Lam *et al* *.*, 2020; Zhang *et al* *.*, 2020c). Environmental samples from the wet market in Wuhan, where the epidemic originated, identified a virus closely related to the isolates from patients. Such a virus might have circulated as a ‘cryptic’ infection for years in the human population before acquiring a critical mutation that started the current pandemic (Zhang and Holmes, 2020).

### Virus mutation

Due to a proof‐reading viral enzyme, the mutation rate of coronaviruses is low for the standards of an RNA virus. Now SARS‐CoV‐2 strains start to differentiate into two subtypes, S and L, with L being newer and now more frequent. There is no indication that this shift is linked to changes in biological properties. SARS‐CoV‐2 is expected to accumulate 1–2 mutation per month during epidemic circulation (Kupferschmidt, 2020). However, clinical samples of COVID‐19 patients from Asia, Australia, Europe and America showed nearly identical RNA sequences (Kim *et al.*, 2020). The few sequence differences can be used for epidemiological analysis. For example, the first Italian COVID‐19 patient in Rome showed a virus sequence closely related to that of a Chinese tourist to Italy while a patient from Lombardy was most closely related to a virus isolated in Munich, suggestive of multiple introduction of SARS‐CoV‐2 into Italy (Giovanetti *et al.*, 2020). RNA sequencing of lung washes demonstrated a median of four viral variants with single nucleotide changes within an individual patient, but only a single variant was transmitted to family members. (Shen *et al.*, 2020b). This study reported another important observation: COVID‐19 patients, when compared with bacterial pneumonia cases, lacked commensal bacteria in the lungs and showed nearly exclusively viral RNA. This indicates massive viral replication in the lung and the absence of a bacterial co‐pathogen.

### Virus excretion

Viral RNA peaked 3 days after symptom onset and decreased over the next 2 weeks. Viral load was higher in the nose than in the throat (Zou *et al.*, 2020). Nasopharynx samples are commonly used for diagnostics, and they are twice as sensitive as oropharynx samples. Obtaining sputum and bronchoscopy samples, which are the most sensitive test material, is linked with substantial infection risk for the physician (Wang *et al.*, 2020a). At admission, mild cases showed a 60‐fold lower respiratory viral load than in severe cases, and they also showed an earlier viral clearance (Liu *et al.*, 2020). Patients from Hong Kong were followed for viral load in self‐collected saliva. Titres were high directly after symptom onset, declined subsequently, but could remain positive for 3 weeks. Titres were higher in older patients, but did not correlate with disease severity. Viruses did not accumulate mutations (To *et al.*, 2020). Patients from Singapore with mild disease demonstrated a median duration of virus shedding of 12 days and half of the patients excreted virus also into the stool (Young *et al.*, 2020). Also, Chinese patients excreted the virus in the stool for up to 12 days; a quarter of the stool samples tested for the virus remained positive, when the respiratory sample had already become negative. Virus replication apparently occurred in the gut, since the virus receptor is also expressed on gut epithelia (Xiao *et al.*, 2020). Faecal virus excretion started after respiratory virus excretion, but remained positive longer than in the throat. Stool virus detection was not associated with diarrhoea (Wu *et al.*, 2020; Wang *et al.*, 2020a).

### Asymptomatic virus excreters

For the understanding and control of viral transmission dynamics in a population, it is important to know whether asymptomatic viral excreters exist. Isolated cases were documented. An asymptomatic adult contact of a patient showed the same viral pattern as the patient (Zou *et al.*, 2020). In a Chinese study, 10% of subjects who tested positive for the virus were symptom free. While most developed symptoms later, a 50‐year‐old woman, infected by her husband in his incubation period, remained healthy despite being virus positive in throat and anal swabs (Luo *et al.*, 2020).Furthermore, a healthy 6‐month baby that contracted the infection from its symptomatic parents; excreted high loads of virus in the nasopharynx for 16 days; showed virus in the blood and stool; but showed no clinical symptoms except for fever for one hour (Kam et al., 2020). Finally, an asymptomatic woman from Wuhan, who was weakly virus‐positive, transmitted the infection to five relatives, two of whom developed severe pneumonia (Bai *et al.*, 2020).

### Viral antibodies

Viral antibody tests become crucial for assessing the degree of viral immunity after an infection, which is of critical importance for planning exit strategies from lockdown situations. All COVID‐19 patients from Hong Kong developed ELISA IgG antibodies against the spike protein, which correlated with neutralizing antibodies (only one subject tested). Antibody titres did not correlate with disease severity (To *et al.*, 2020). It is safer to evaluate protective immunity based on neutralizing antibody determination than on antibodies determined with ELISA. The cell line VeroE6 was engineered to express the protease TMPRSS2, which assists viral entry into the cell, resulting in efficient infection of the cell line and production of high yields of SARS‐CoV‐2. This cell line is therefore also useful for the testing of neutralizing antibody titres in serological surveys and in vaccination trials (Matsuyama *et al.*, 2020).

### Spike protein structure

The coronavirus spike protein was intensively investigated because this knowledge will be crucial for vaccine development. One group determined the 3D structure of the spike protein from SARS‐CoV‐2 by cryo‐electron microscopy. It differs from SARS‐CoV (the coronavirus from the 2002 SARS epidemic, sharing the same cell receptor ACE‐2) over the receptor‐binding domain (RBD), allowing tighter binding to ACE‐2. Monoclonal antibodies directed to SARS‐CoV do not bind well to SARS‐CoV‐2, underlining distinct antigenicity. The proteolytic cleavage site (important for the fusion process mediating virus cell entry) is also different (Wrapp *et al.*, 2020). The viral spike protein presents its receptor‐binding domain (RBD) in two conformations, down and up. In its up position, RBD binds to the N‐terminal domain of the ACE‐2 dimer (Yan *et al.*, 2020). The crystal structure of viral spike‐human receptor interaction was described by another group as a concave viral cup with a protruding ridge embracing closely the exposed N‐terminal end of the human ACE‐2 receptor. The novel coronavirus shows a larger binding interfacing, and more contacts to the receptor, than SARS‐CoV. The spike protein of a closely related bat coronavirus also mediated entry into cells via the ACE‐2 receptor. However, the bat spike protein was not cleaved upon entry, in contrast to SARS‐CoV‐2 spike protein, which contains a target site for the protease furin (Lan *et al.*, 2020; Shang *et al.*, 2020). A panel of mouse monoclonal antibodies, or neutralizing polyclonal antibodies directed against SARS‐CoV spike protein, failed to recognized the SARS‐CoV‐2 spike protein, again demonstrating distinct epitope structures between the two viruses (Wang et al., 2020b). Due to amino acid and glycosylation differences between both viruses, a neutralizing monoclonal antibody to SARS‐CoV binds only with 100‐fold lower affinity to the viral protein and lacks neutralizing activity against SARS‐CoV‐2 (Yuan et al., 2020).

### Antivirals

Two approaches, which were already used against SARS‐CoV, were also tested against SARS‐CoV‐2. A possible drug target is the protease of SARS‐CoV‐2, which cleaves the polyprotein translated from the viral RNA genome into the mature viral proteins. Medicinal chemists modified a ketoamide inhibitor, active against SARS and MERS viruses such that it also inhibited SARS‐CoV‐2 at micromolar concentrations, as demonstrated by *in vitro* infection tests of lung cells. By subcutaneous and inhalation application, the inhibitor reached the lungs of mice in meaningful pharmacological concentrations (Zhang et al., 2020a).

Human recombinant soluble ACE2 protein reduced the infection of Vero cell cultures and of organoids, with SAR‐CoV‐2 in a dose‐dependent way. Soluble ACE‐2 has two possible modes of action: first as a receptor decoy. Second, since ACE2 is a lung protection factor which is removed from the cell membrane during virus entry, a soluble ACE‐2 could also confer increased lung protection. This concept was developed for SARS, and the soluble ACE‐2 protein has already undergone clinical safety trials, but it is unknown whether it has clinical efficacy (Monteil *et al.*, 2020).

## Conclusion

At the moment of the writing, we are still in the rising phase of the COVID‐19 pandemic, where we do not know where the human death toll (currently 161’000 victims) will finally level off. The economical and societal consequences cannot yet be assessed. The virus might well change our societies. If one lesson is already clear to the author of these lines, it is that it is time to create the infrastructure for a World Health Organization worthy of its name. The impact of the current pandemic shows that we cannot expect guidance and rescue from an organization having a budget only equal to that of the University Hospital of Geneva (2.5 vs. 1.9 billion $, respectively), which must merely serve a canton in a small country. Retrospectively, it is apparent that we have invested in the wrong societal insurance, military defence, but not defence of our health, and so we are now paying a heavy human and economic price for our limited foresight.

## Conflict of interest

The author consults Nestlé, his former employer, on the scientific aspects of the COVID‐19 epidemic, but he does not consider this as a conflict of interest.
